# Identification of SNPs in Closely Related *Temperate Japonica* Rice Cultivars Using Restriction Enzyme-Phased Sequencing

**DOI:** 10.1371/journal.pone.0060176

**Published:** 2013-03-26

**Authors:** Sang-Ic Kim, Thomas H. Tai

**Affiliations:** USDA-ARS Crops Pathology and Genetics Research Unit, Department of Plant Sciences, University of California Davis, Davis, California, United States of America; CNR, Italy

## Abstract

Very low polymorphism in the germplasm typically used by breeding programs poses a significant bottleneck with regards to molecular breeding and the exploitation of breeding materials for quantitative trait analyses. California rice cultivars, derived from a very small base of *temperate japonica* germplasm and having a relatively brief breeding history, are a good example. In this study, we employed a reduced representation sequencing approach called Restriction Enzyme Site Comparative Analysis (RESCAN) to simultaneously identify and genotype single nucleotide polymorphisms (SNPs) in forty-five rice cultivars representing the majority of the 100 year-old breeding history in California. Over 20,000 putative SNPs were detected relative to the Nipponbare reference genome which enabled the identification and analysis of inheritance of pedigree haplotypes. Haplotype blocks distinguishing modern California cultivars from each other and from the ancestral short grain *temperate japonica* cultivars were easily identified. Reduced representation sequencing methods such as RESCAN are a valuable alternative to SNP chip genotyping and low coverage whole genome sequencing.

## Introduction

Recent advances in genomics have made single nucleotide polymorphisms (SNPs) the marker of choice for genome-wide genetic analyses. Since SNPs occur at much higher density than other markers such as microsatellites, they are particularly useful in distinguishing closely related individuals. In rice (*Oryza sativa* L.), the sequencing of the *temperate japonica* cultivar Nipponbare [1] established a gold standard reference for the identification of SNPs using expressed sequence tags and other genome sequence data (e.g. *indica* 93-11, [2]). Resequencing of several different accessions representing the five major subpopulations of cultivated Asian rice [3], [4] has facilitated the development of SNP microarrays or chips for analyses of both diverse germplasm accessions for genome-wide association studies [5], [6] and closely related cultivars to facilitate the genetic analyses of and more fully exploit the phenotypic variation that currently exists in elite breeding programs [4]. Whole genome sequencing has also been employed directly to genotype rice germplasm for genome-wide association analyses and to dissect the origins of cultivated rice [7]–[9].

A number of other powerful, next-generation sequencing-based methods for the simultaneous identification, confirmation, and genotyping of SNPs are now being employed in model and non-model species [10]–[12]. One general approach developed over a decade ago for use with Sanger sequencing [13] involves construction of sequencing libraries with reduced genomic complexity (i.e. reduced representation sequencing). Several methods coupled with various next-generation sequencing platforms have been developed and successfully employed in recent years [14]–[19]. By reducing the complexity of a sequencing library, the coverage (i.e. number of times a given fragment is sequenced) is increased, thus increasing the confidence with which a putative SNP can be called. Reduced complexity or representation in combination with the use of unique DNA barcoded adapters for each genotype allows multiplexing of libraries from several individuals to exploit the enormous capacity of next-generation sequencing platforms.

The history of California rice breeding is relatively short (∼100 years) and most of the modern cultivars can be traced back through their pedigrees to 23 ancestral introductions [20]. Prior to the establishment in 1969 of an accelerated rice breeding program [21], three major cultivars, Colusa (1917), Caloro (1921), and Calrose (1948), were grown in California. These were tall plant types with short or medium grains; medium grain rice remains the predominant grain type in California to this day. In the mid-1970’s to mid-1980’s, the semidwarf trait was incorporated into California rice cultivars through induced mutation of Calrose resulting in Calrose 76 [22] and the introgression of the *sd-1* locus from the *indicas* IR-8 and Taichung Native-1 during the breeding of the cultivars M9 [23] and L-202 [24] respectively. While the short and medium grain cultivars have been developed primarily through crossing within the *temperate japonicas* and induced mutation of adapted cultivars, the long grain California cultivars (including aromatic and specialty rices) have been developed with more diverse germplasm as reflected in the pedigrees (Table S1).

Given the extremely narrow germplasm base of California cultivars, particularly the short and medium grain types, we sought to employ a genotyping by sequencing approach to evaluate forty-five cultivars released from 1917–2006. We employed a reduced representation sequencing method called Restriction Enzyme Site Comparative Analysis (RESCAN), which has been previously used in rice and *Arabidopsis* [19], [25], in order to simultaneously discover and genotype SNPs. We identified over 20,000 putative SNPs with respect to the Nipponbare reference genome. Using a subset of ∼4,500 SNPs, we were able to define pedigree haplotypes, examine their origins, and demonstrate the utility of this genotyping approach for overcoming bottlenecks to molecular breeding and quantitative trait analysis resulting from the extremely reduced diversity commonly found in elite breeding programs.

## Results and Discussion

### Construction of RESCAN libraries

**Figure 1:**
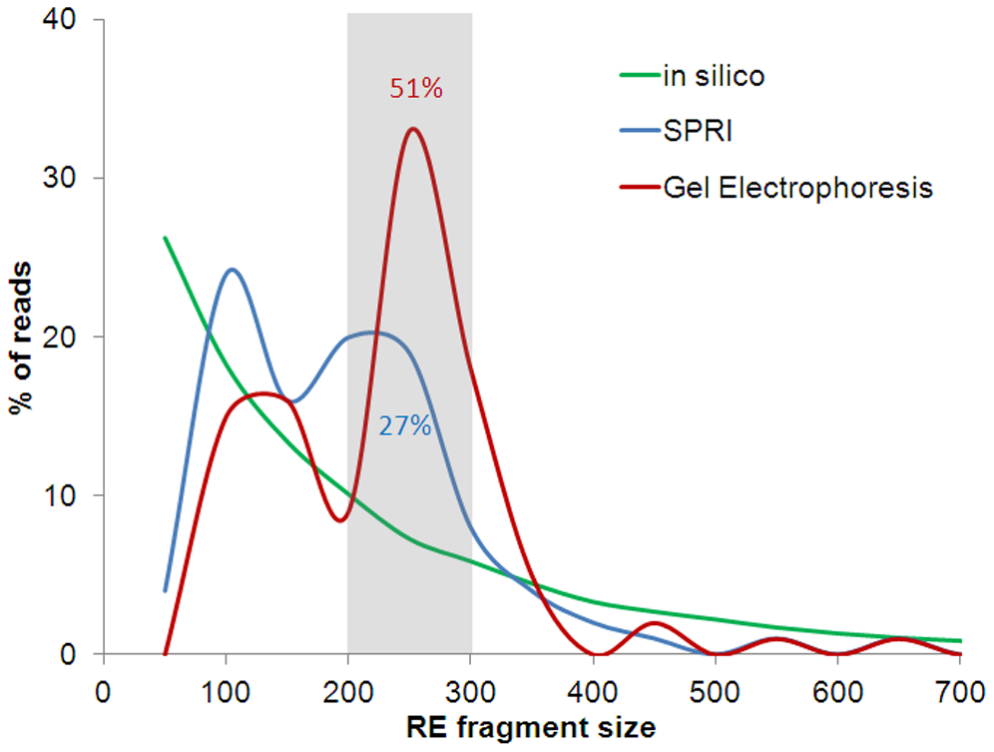
Distribution of RESCAN reads generated from two size selection methods. *In silico* NlaIII digestion of total Nipponbare genome (black line) showed that about 13% of total restriction fragments exist in the range of target size (200–300 bp). About 51% of the reads from the gel electrophoresis (LabChip) method were in the target range compared to 27% of the reads from the SPRI (AMPure) method.

A simple, robust method of constructing sequencing libraries of reduced genomic complexity was employed to facilitate simultaneous SNP discovery and genotyping through multiplexing of forty-five California rice cultivars (Table S1). This strategy called RESCAN [19] combines digestion using a restriction endonuclease with a 4 bp recognition site, ligation with unique barcoded adapters, and size fractionation of pooled ligation products to produce libraries for Illumina short read sequencing. In this case, we used the restriction enzyme NlaIII (5’-G/ATC-3’) which generates approximately 1.8 M fragments based on *in silico* digestion of Nipponbare reference genome [19]. Fragments in the range of 350–450 bp, corresponding to 200–300 bp prior to ligation (∼240,000 fragments, 12.85% of the genome), were size selected using two strategies. The first involved using solid phase reversible immobilization (SPRI, [26]) paramagnetic beads in a two-step double-SPRI protocol [19], [27]. In the second strategy, we employed an automated gel electrophoresis fractionation system (LabChip®XT, Caliper Life Sciences, Hopkinton, MA). The accuracy of these methods was validated by the proportion of sequenced fragments which was targeted to the predicted size fractions based on the *in silico* digestion of the Nipponbare reference genome. While both fractionation methods significantly enriched the fragments in the target size range, the electrophoresis fractionation method produced a greater effect than the SPRI-based protocol (4-fold vs. 2-fold enrichment; Fig. 1).

### SNP detection and genotyping by sequencing

The multiplexed library pools from each size fractionation method were sequenced on single lanes of an Illumina HiSeq2000 (Illumina, San Diego, CA). A total of ∼240 M reads (∼12 Gb raw sequence reads) were obtained from the two runs (150 M reads for gel electrophoresis and 94 M reads for SPRI). After de-multiplexing according to unique barcodes for each genotype and filtering bad sequences, a combined total of 208 M reads (138 M reads for gel electrophoresis and 70 M for SPRI) were obtained (Table 1). Combined sequencing reads for each cultivar ranged from 0.9 M (0.4%) to 8.5 M (3.7%) with a mean of 4.63 M±2.18 M SD (Table S2). About 97% of the sequences could be aligned to the Nipponbare genome. SNPs were identified based on the sequence discrepancies between each cultivar and Nipponbare. A total of 20,987 SNPs were identified which is an average of 0.06 SNP per kb or one SNP in every 16 kb of the rice genome (Table S3). In contrast, 0.17 SNPs per kb were detected from the whole genome sequencing and comparison of Koshihikari with the Nipponbare reference genome [4] and 9.32 SNPs per kb obtained from sequencing 517 rice landraces [7]. Differences in the SNP density in our study may be due to the reduction of genomic complexity, the sequence coverage, or the genetic relatedness of the cultivars and Nipponbare. Of the 20,987 SNPs detected, missing data across the 45 cultivars ranged from 40% to 96% with a mean of 70%±17% SD.

**Table 1:** Sequencing results for forty five California cultivars.

No.	Cultivar	Total reads	Aligned reads	Sequenced (Mb)	SNPs	SNPs/sequenced 100 kb
1	Colusa	8,909,990	8,355,240	16.7	861	5.2
2	Caloro	5,571,659	5,110,290	10.2	610	6.0
3	Calrose	6,418,466	6,234,660	12.5	1,887	15.1
4	CS-M3	6,313,437	5,741,670	11.5	1,910	16.6
5	CS-S4	4,157,417	4,111,110	8.2	498	6.1
6	M5	9,117,640	8,406,930	16.8	2,733	16.3
7	S6	6,459,490	5,966,973	11.9	1,429	12.0
8	Calrose76	5,772,255	5,473,152	10.9	1,417	12.9
9	M7	3,090,181	2,893,575	5.8	777	13.4
10	M9	4,389,869	4,324,590	8.6	1,859	21.5
11	Calmochi-201	2,977,348	2,799,468	5.6	541	9.7
12	L-201	8,248,291	7,832,565	15.7	7,422	47.4
13	M-101	7,284,788	6,607,359	13.2	1,756	13.3
14	M-301	6,928,960	6,401,370	12.8	2,017	15.8
15	S-201	8,730,052	8,398,140	16.8	2,095	12.5
16	Calmochi-202	4,122,284	3,840,480	7.7	768	10.0
17	M-302	7,000,246	6,561,687	13.1	2,132	16.2
18	M-401	3,637,833	3,322,450	6.6	835	12.6
19	M-201	2,091,366	1,963,395	3.9	721	18.4
20	L-202	2,165,942	1,974,560	3.9	877	22.2
21	Calmochi-101	1,898,160	1,772,280	3.5	215	6.1
22	M-202	2,359,219	2,131,800	4.3	542	12.7
23	A-301	4,734,314	4,260,360	8.5	2,932	34.4
24	M-102	6,961,247	6,444,620	12.9	2,700	20.9
25	M-203	4,919,289	4,527,200	9.1	1,531	16.9
26	S-101	4,457,206	4,263,336	8.5	1,639	19.2
27	M-103	2,136,715	2,070,880	4.1	268	6.5
28	S-301	2,083,500	2,006,825	4.0	394	9.8
29	L-203	2,208,945	2,029,374	4.1	990	24.4
30	M-204	5,056,909	4,868,400	9.7	2,505	25.7
31	A-201	8,538,074	7,685,760	15.4	6,776	44.1
32	L-204	3,954,634	3,545,600	7.1	1,569	22.1
33	S-102	4,590,403	4,337,830	8.7	1,132	13.0
34	Calhikari-201	3,801,227	3,641,190	7.3	856	11.8
35	Calmati-201	3,367,693	3,300,425	6.6	2,276	34.5
36	L-205	954,074	921,403	1.8	206	11.2
37	M-402	2,162,900	1,948,150	3.9	483	12.4
38	M-104	2,976,145	2,897,160	5.8	904	15.6
39	M-205	2,343,036	2,270,268	4.5	448	9.9
40	M-206	5,476,784	5,189,895	10.4	2,092	20.2
41	M-207	4,194,550	3,859,123	7.7	1,378	17.9
42	Calamylow-201	3,879,058	3,592,160	7.2	840	11.7
43	Calmati-202	1,891,807	1,700,250	3.4	512	15.1
44	L-206	4,566,763	4,270,805	8.5	2,746	32.1
45	M-208	5,430,022	5,211,900	10.4	1,635	15.7
	Sum	208,330,188			20,987	

In order to assess the SNP scoring error rate, CAPS markers were designed from SNPs identified from RESCAN (Table S4) and used to re-score the cultivars. Based on the analysis of five CAPS markers, the error rate was calculated to be less than 2% (Table S5). About 61% of SNPs were found in genic regions (exons–33%, introns–22%, 5′ UTRs–2%, and 3′ UTRs–4%; Fig. S1). The proportion of SNPs found in the genic regions is similar to those reported by McNally et al. [3]. All SNPs were fairly well-distributed over the genome, ranging from 32.5 SNPs per Mb on chromosome 2 to 111 SNPs per Mb on chromosome 11 (Fig. S2). The results show that the genome-wide distribution of SNPs derived from RESCAN was suitable for defining pedigree haplotypes and genetic relationships in the very closely related California cultivars.

Since more SNPs were found in cultivars with greater sequence coverage (both in total reads and reads per given SNP) the frequency of SNPs in each cultivar was compared by using the total SNPs per sequenced 100 kb (Table 1). Colusa and Caloro, which are the two oldest California cultivars and the ancestors of the short and medium grain rice cultivars that dominate the California industry, had the fewest SNPs indicating that they are most closely related to Nipponbare. L-201 had the highest frequency of SNPs (47.4 SNPs/sequenced 100 kb). The most SNPs (28.7 SNPs/sequenced 100 kb) were found in the *tropical japonica* (long grain) and the fewest SNPs (10.2 SNPs/sequenced 100 kb) were found in *temperate japonica* short grains while 15.6 SNPs/sequenced 100 kb were found in *temperate japonica* medium grains. These results are consistent with the pedigrees of the cultivars. More diverse germplasm has been incorporated in the breeding of the long grain California cultivars with contributions from the southern U.S. rice belt breeding programs which primarily focus on long grain types. On the other hand, the short and medium grain California cultivars share more common ancestors and the lower density of SNPs in the short grain cultivars likely reflects the contributions of Japanese rice germplasm in the breeding of these types.

**Figure 2:**
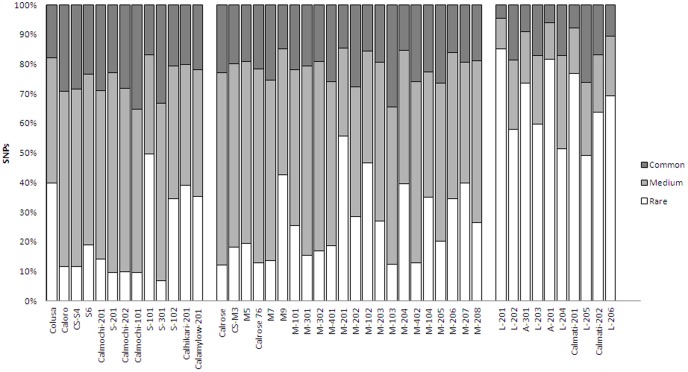
Composition of SNP types in California cultivars. SNPs were grouped into three categories: rare SNPs found in less than 15 cultivars, medium frequency SNPs found in between 16–30 cultivars and common SNPs found in more than 30 cultivars.

Some SNPs were only found in certain cultivars while others were common to most of the California cultivars. About 9% of the SNPs that were detected were found in only one cultivar while no SNP was present in all 45 cultivars. In particular, L-201 (32%), A-201 (22%) and Calmati-201 (23%) contained a high proportion of unique SNPs. In order to compare the type of SNPs in California cultivars, we grouped SNPs into three categories: rare SNPs found in less than 15 cultivars, medium frequency SNPs found in between 16–30 cultivars and common SNPs found in more than 30 cultivars. The composition of each group in California cultivars were presented in Fig. 2. All the long grain cultivars have high portion (more than 50%) of rare SNPs. In contrast, only S-101 from the short grain types and M-201 from the medium grain types have 50% of rare SNPs. Identified SNPs were grouped into three categories based on the frequency with which they are detected in the 45 California cultivars (Fig. 2).

**Figure 3:**
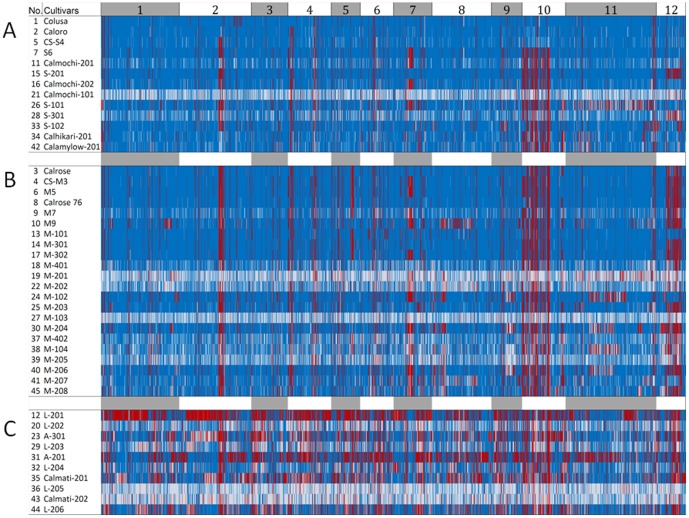
High-density haplotype maps of 45 California cultivars generated by RESCAN. (A) short grain (B) medium grain (C) long grain Regions in blue are monomorphic with regard to the Nipponbare reference genome. Regions in red represent haplotype blocks and white areas represent missing data.

High-density haplotype maps were generated based on the SNP data (Fig. 3). Highly conserved blocks were detected in chromosomes 10 and 2 that were not present in Nipponbare or either of the two ancestral short grains Colusa and Caloro from which the modern short and medium grain California cultivars are largely derived. The blocks in chromosome 10 were also absent in CS-S4 which is derived primarily from Caloro. A review of the pedigrees (Table S1) reveals that the Louisiana cultivar Lady Wright (via Calady) is also an ancestor of the modern short and medium grain cultivars, although not of CS-S4, and may be the source of the variation in chromosome 10. The contribution of other breeding germplasm from the southern U.S. rice belt origin could explain the presence of the chromosome 10 haplotype blocks in the long grain California cultivars. Lady Wright also appears to be the most likely source of the variation observed in chromosome 2 based on the presence of the haplotype blocks in Calrose but not Caloro. However, unlike the chromosome 10 blocks, the conserved region in chromosome 2 is found in CS-S4 which does not have Lady Wright in its pedigree. CS-S4 was developed from a series of crosses between Caloro and selections derived from a cross between Caloro and Smooth No. 3 [28]. Smooth No. 3, a glabrous accession of unknown origin, is thus the likely source of the chromosome 2 blocks in CS-S4 and suggests a relationship with Lady Wright and other southern U.S. rice breeding germplasm. Whether the regions in chromosome 10 and 2 harbor genes controlling traits important for production in the Mediterranean environment of California and reflects selections by breeders remains to be determined.

### Genetic diversity among California cultivars

**Figure 4:**
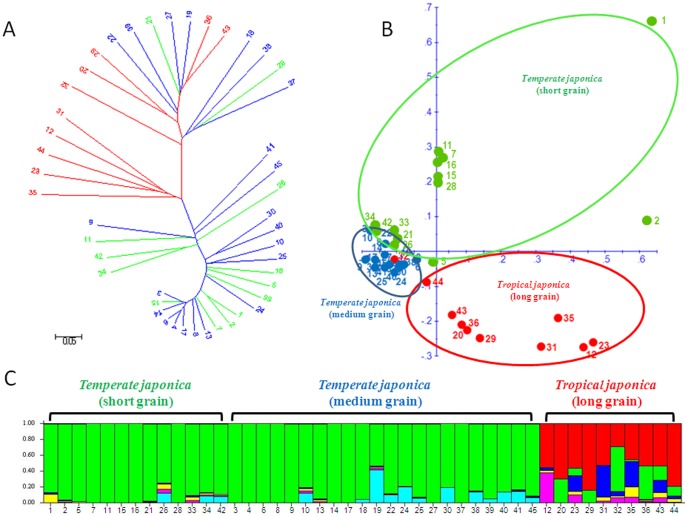
Phylogenic analysis of 45 California cultivars. (A) Unrooted neighbor-joining tree showing genetic relationships among the cultivars based on C.S. Chord distance calculated using 4,469 SNPs (B) Principal-coordinates analysis (PCA) plot showing that *temperate japonica* medium grain group is clearly distinguished from *tropical japonica* group. The X and Y-axes show PC1 and PC2, respectively, of the PCA analysis of the SSR data. (C) Population structure analysis plots (K = 4) generated using the STRUCTURE program. The colors of the numbers represent the respective groups: red, *temperate japonica* (short grain); black, *temperate japonica* (medium grain); blue, *tropical japonica* (long grain) respectively.

For phylogenetic analysis, 4,469 SNPs were selected which were scored in 23 or more of the cultivars. Missing data for this subset of SNPs ranged from 1.5% to 85.5% with a mean of 31%±26% SD. Two distinct groups are observed in the resulting neighbor-joining tree, which is consistent with the two *japonica* subgroups of rice (*temperate* and *tropical*; Figure 4A). Within the *temperate japonica*, short grain and medium grain groups were clustered. Early California cultivars (i.e. Colusa, Caloro, Calrose, CS-M3, and CS-S4) clustered closely regardless of their grain size. Principal component analysis (PCA) also indicated most of the short grain *temperate japonica* accessions were separated from others while the medium grain *temperate japonicas* clustered closely (Fig. 4B). With the exception of L-204, the *tropical japonicas* (nine out of ten cultivars) clustered together.

Population structure analysis was conducted using the STUCTURE program from K (number of fixed subgroups or clusters) values ranging from 1 to 10. Separation of the *temperate* and *tropical japonica* subgroups was observed starting at K = 2. However, short and medium grain groups were indistinguishable even at the highest K values. The likelihood value decreased continuously and the most significant change was observed between K = 4 and K = 5 (Fig. S3). Population structure at K = 4 indicated that each subgroup can be classified into two groups with admixture in each group (Fig. 4C). While the population-differentiation statistics (*Fst*) values between major subgroups were 0.85, the differences within the subgroup were very small (less than 0.01) suggesting that California cultivars can be divided into two major groups.

### Assessment of California pedigrees

**Figure 5:**
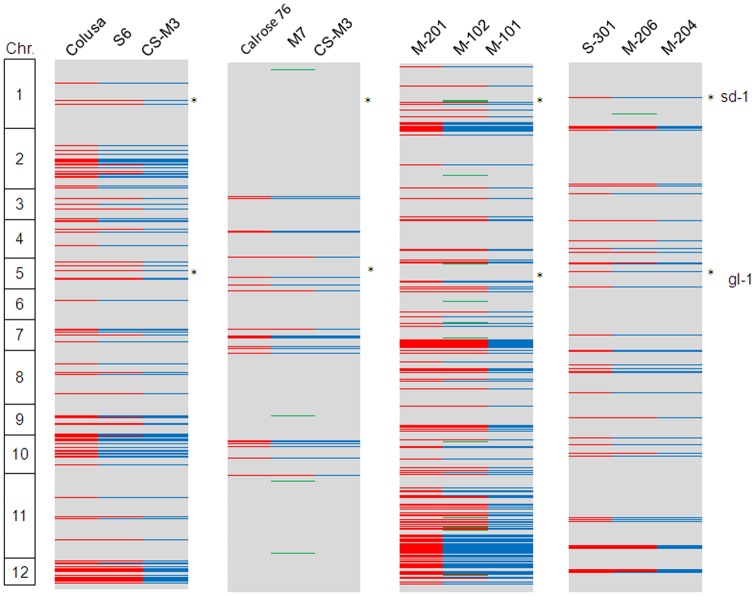
Evaluation of pedigrees of California cultivars derived from bi-parental crosses. Grey areas represent SNPs shared the parents and the descendent cultivars. Red areas represent haplotype blocks shared with the female parent blue areas represent those shared with the male parent. Green areas are haplotypes that are not derived from either parent and may represent scoring errors or differences between the accessions used in this study and those used originally for the breeding of the cultivars. The semidwarf-1(*sd-1*) and glabrous-1 (*gl-1*) loci are indicated with an asterisk (*).

Four of the California cultivars were derived from crosses between two other cultivars (Table S1). S6 was developed from ‘Colusa’/’CS-M3’, M7 was developed from ‘Calrose 76’/‘CS-M3’, M-102 was developed from ‘M-201’/‘M-101’and M-206 was developed from ‘S-301’/‘ M-204’. The SNP data clearly identified chromosome segments in each cultivar that appeared to be derived from one or the other parent (Fig. 5). In the case of the short grain cultivar S6, its overall phenotype more closely resembles Colusa than CS-M3 [29]. Of the SNPs detected between S6 and Nipponbare, 86.5% were shared with both parents while of the remaining SNPs, 8.5% were in common with CS-M3, 4.9% were in common with Colusa, and 0.1% could not be traced back to either parent. Although M7 resembles CS-M3 more closely than Calrose 76 in overall phenotypic characteristics some traits clearly appear to originate from a particular parent. For example, CS-M3 is tall and has glabrous leaves and hulls while Calrose 76 is short-statured and has pubescent leaves and hulls. M7 is short-statured and glabrous. Interestingly, over 96% of the SNPs detected between M7 and the reference Nipponbare genome were monomorphic with the parental cultivars and only ∼4% of SNPs could be assigned to one of the parents (0.6% from Calrose76 and 2.8% from CS-M3) while 0.6% could not be assigned to either parent. The *sd-1* introgression from Calrose 76, which is presumably responsible for the short stature of M7, could not be detected. However, an introgression from CS-M3 on chromosome 5 for the glabrous locus (*gl-1*) was found in M7. Of the four cultivars, M-102 exhibited the highest proportion of SNPs that appeared to originate from one parent with 11.6% SNPs shared with M-201 and 11.5% shared with M-101. M-102 also had the highest percentage of unexplained SNPs at ∼2%. The SNP data indicated that M-102 has the Calrose 76 *sd-1* allele from M-101 (M-201 has the IR8 *sd-1* allele) which was consistent with previous analysis of the *sd-1* alleles present in California cultivars [30]. M-206 is the most recently developed of the four cultivars. Only 7.2% of SNPs in M-206 could be assigned to one of the parents (2.8% from S-301 and 3.7% from M-204) while 0.6% of the SNPs were unexplained. Like M-102, M-206 has the Calrose 76 *sd-1* allele from S-301 parent [30], which is reflected by the SNP data. The percentage of unique SNPs which are not matched to either parent was fewer than 2% in four cultivars which is similar to SNP scoring error rate.

### Efficiency of the RESCAN method

As with other genotyping by sequencing methods [10], [12], [18], we observed high levels of missing data for a number of individuals due to low sequence coverage (Table S3). This issue can be addressed directly by increasing the coverage or indirectly through imputation of missing genotypes [18], [31]. Greater sequence coverage can be achieved by adjusting parameters for complexity reduction. As noted earlier, we selected a size range of 200–300 bp (size prior to ligation of adapters) which consists of ∼240,000 *NlaIII* fragments based on *in silico* digestion of the Nipponbare genome. By raising the size range to 300–400 bp (∼140,000 fragments), 500–600 bp (∼54,000), or 700–800 bp (24,269), a significant reduction in target sequences and therefore greater sequence coverage can be achieved. It should also be noted that improvements in Illumina HiSeq2000 sequencing have increased the numbers of reads to approximately 2-fold (currently >300 million reads per lane). In this study, individuals with ∼2.5–3 M reads had ∼50% or fewer missing data. By employing a 300–400 bp size fractionation window, the current Illumina HisSeq2000 output should provide sufficient coverage of a multiplex of 96 individuals per lane for a comparable level of missing data. At these levels of multiplexing, RESCAN may be applied cost-effectively to linkage analysis of single genes and QTLs as well as genome-wide association studies and genomic selection [12]. Other strategies to increase sequence coverage include re-sequencing under-represented libraries or reducing multiplexing levels. However, missing data may be addressed in a more efficient and cost-effective manner through genotype imputation [32], which is of particular value in studies involving genome-wide association analysis or fine-mapping. In rice, substantial SNP data from re-sequencing and SNP microarray studies [3]–[6] are available for use in genotype imputation although most of these studies have focused on diverse germplasm. Andolfatto et al. [18] demonstrated the efficacy of imputation in conjunction with reduced representation sequencing-based genotyping method which is very similar to RESCAN.

## Conclusions

The RESCAN method facilitated the rapid, cost-effective discovery and genotyping of thousands of SNPs in very closely related California *temperate japonica* cultivars. Our results were consistent with the pedigree information and we were able to examine the origin of various haplotype blocks defined by the RESCAN SNPs as well as to identify regions that may have undergone selection for rice production in the Mediterranean climate of California. In addition to defining introgressions in the cultivars examined, the density of SNPs detected by RESCAN in this study was sufficient for single gene and QTL mapping. Like other genotyping by sequencing approaches, the RESCAN method is amenable to commonly employed strategies to increase marker density for applications such as genome-wide association studies and genomic selection.

## Materials and Methods

### Reduced representation sequencing of rice cultivars

To facilitate the identification of SNPs, a reduced representation sequencing strategy called Restriction Enzyme Site Comparative Analysis (RESCAN, [19]) was employed. The RESCAN method involves generation and pooling of restriction enzyme-phased DNA libraries which are subjected to size fractionation followed by Illumina short read sequencing. RESCAN library construction is simple, inexpensive, and robust.

#### Rice cultivars

Forty-five California rice cultivars were used in this study (Table S1). Seeds were obtained from the California Cooperative Rice Research Foundation Rice Experiment Station (Biggs CA). DNA was isolated from leaf tissues of ∼20 three week-old seedlings per cultivar as previously described [30].

#### Construction and sequencing of RESCAN libraries

RESCAN libraries were constructed as described previously [19] with some modifications. For each cultivar, 100 ng of genomic DNA was digested with the restriction enzyme NlaIII (CATG↓; NEB, Ipswich, MA) for 1 hour at 37°C. Completion of digestion was verified on agarose gel electrophoresis and digestions were stopped and purified using 0.8X AMPure SPRI beads according to manufacturer's instructions (Beckman Coulter Genomics, Danvers, MA). The DNAs were ligated with unique barcoded adapters based on the Y-adapter system used for standard Illumina sequencing library construction and formed by two oligonucleotides (e.g., adA2_NlaIII_01_aaaca: 5′-P-aaacaAGATCGGAAGAGCGGTTCAGCAGGAATGCCGAG-3′; adB2_NlaIII_01_aaaca: 5′-ACACTCTTTCCCTACACGACGCTCTTCCGATCTtgttt **CATG**-3′. NlaIII site in bold and 5-bp barcode underlined). Sequences of barcodes used in this study are shown in Table S2. Ligations were performed using a Quick Ligation kit (NEB) according to manufacturer's instructions. Reactions were stopped and purified using 0.8X AMPure SPRI beads, quantified by fluorescent measurement using the Quant-it™ Picogreen® DNA assay (Invitrogen, Carlsbad, CA), and pooled in equal amounts (total of ∼1 µg) prior to size fractionation. For size fractionation, two different methods were employed to select fragments primarily in the range of 350–450 bp: (1) double size selection with AMPure SPRI beads as described by Monson-Miller et al. [19]; (2) gel electrophoretic selection using a LabChip® XT system and DNA 750 Assay kit according to manufacturer's instructions (Caliper Life Sciences; Mountain View, CA). Size-selected libraries were then enriched by PCR (15 cycles) using standard Illumina PE1 and PE2 primers and Phusion HF Master Mix (NEB). PCR products were purified using 0.8X AMPure beads. The quality of the libraries were checked by size range analysis using a DNA 1000 Assay on a 2100 Bioanalyzer (Agilent Technologies) and quantified by fluorescent measurement using the Quant-it™ Picogreen® DNA assay (Invitrogen). Sequencing was performed at the UC Davis Genome Center Core Service Facility using a single lane of an Illumina HiSeq2000 (Illumina, San Diego, California) with 50 bp single-end reads.

#### Computational analysis and SNP detection

Illumina single-end 50 bp sequence reads were trimmed for adapter contamination and for quality using Scythe and Sickle (https://github.com/ucdavis-bioinformatics). The quality was checked at each step using the Quick Read Quality Control R package (http://www.bioconductor.org/packages/2.10/bioc/html/qrqc.html). Individual libraries (i.e. cultivars) were identified by their unique barcode. The reads were then aligned to the *O. sativa* 'Nipponbare' genome using BWA [33]. The resulting BAM alignment files were sorted and RG tags were added in order to facilitate the variant calling step. The "samtools pileup”, "bcftools view" and "vcfutils.pl varFilter" commands (using a max depth of 1000) were used in a pipeline to call the SNPs (SAMTOOLS [34]). Next, a custom script developed by the UC Davis Genome Center Bioinformatics Core Services was used to filter the SNPs based upon a minimum overall quality of 20, a minimum per sample depth of 4, a minimum conditional genotype quality of 20, a minimum number of significant variants across all samples, and having at least two to three different genotypes across all the samples. The resulting SNPs were spot-checked by looking at the read alignments in Integrative Genomics Viewer (http://www.broadinstitute.org/igv/) for the samples.

#### Cleaved Amplified Polymorphic Sequences (CAPS) marker analysis

Specific primers (Table S4) were designed about 150–200 bp upstream and downstream of the SNPs which are recognized by NlaIII (CATG↓). Fragments of about 350 bp were amplified by PCR. PCR reactions were performed in 25 µl reactions containing 50 ng of genomic DNA, 100 nM primer, and 12.5 µl of 2X Phusion HF Master Mix (NEB) using PCR conditions of 95°C for 10 min and 40 cycles at 95°C for 15 s; 60°C for 1 min. Purified PCR products were incubated with NlaIII (NEB) for 1 hr and run on a 2% agarose gel. CAPS marker scoring was based on differences in fragment sizes compared to Nipponbare genomic DNA (Table S5).

#### Phylogenetic analysis

Genetic distance was calculated based on the neighbor-joining method using the C.S. Chord distance [35] implemented in PowerMarker program version 3.25 (http://www.powermarker.net). The MEGA5 program [36] was used to construct an unrooted neighbor-joining tree. Population structure was analyzed using the model-based program STRUCTURE [37], [38]. The number of clusters (K) from 1 to 10 was calculated by averaging three independent runs with a burn-in period of 5,000.

## Supporting Information

Figure S1 Distribution of SNPs on the annotated gene structures.
(DOCX)

Figure S2 Chromosomal distribution of SNPs found in 45 California cultivars.
(DOCX)

Figure S3 Population structure analysis using the STRUCTURE program.
(DOCX)

Table S1 List of California rice cultivars.
(DOCX)

Table S2 Multiplexing barcodes and number of raw sequence reads.
(DOCX)

Table S3 List of the 4,469 SNPs used for the phylogenetic analysis.
(XLSX)

Table S4 Primer sequences for CAPS marker.
(DOCX)

Table S5 Comparison of genotype results between RESCAN and CAPS marker.
(DOCX)
