# Supplementary figures and images for: Identification of SNPs in Closely Related Temperate Japonica Rice Cultivars Using Restriction Enzyme-Phased Sequencing
Source: PLoS One. 2013 Mar 26;8(3):e60176. doi: 10.1371/journal.pone.0060176 (PMC3608622; doi:10.1371/journal.pone.0060176)

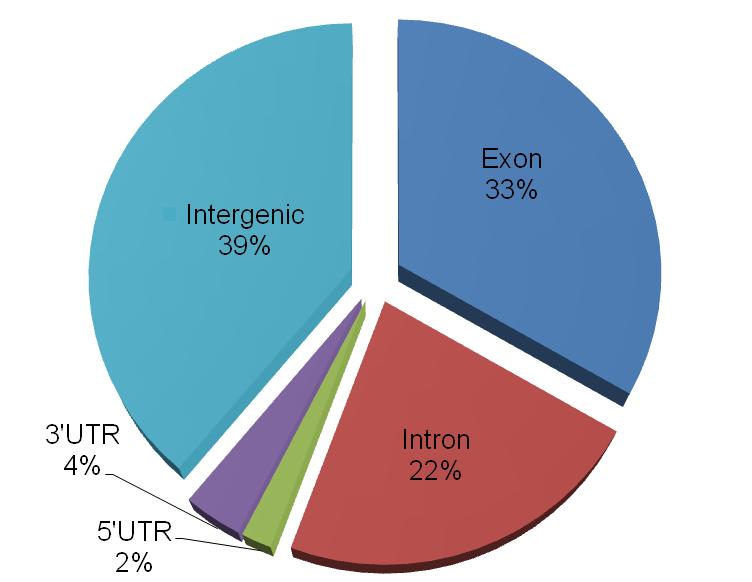


**Fig. S1 Distribution of SNPs on the annotated gene structures**

Supplement: Figure S1 — Distribution of SNPs on the annotated gene structures. (DOCX) [file pone.0060176.s001.docx]
