# Supplementary material for: Identification of SNPs in Closely Related Temperate Japonica Rice Cultivars Using Restriction Enzyme-Phased Sequencing
Source: PLoS One. 2013 Mar 26;8(3):e60176. doi: 10.1371/journal.pone.0060176 (PMC3608622; doi:10.1371/journal.pone.0060176)
Supplement: Figure S2 — Chromosomal distribution of SNPs found in 45 California cultivars. (DOCX) [file pone.0060176.s002.docx]

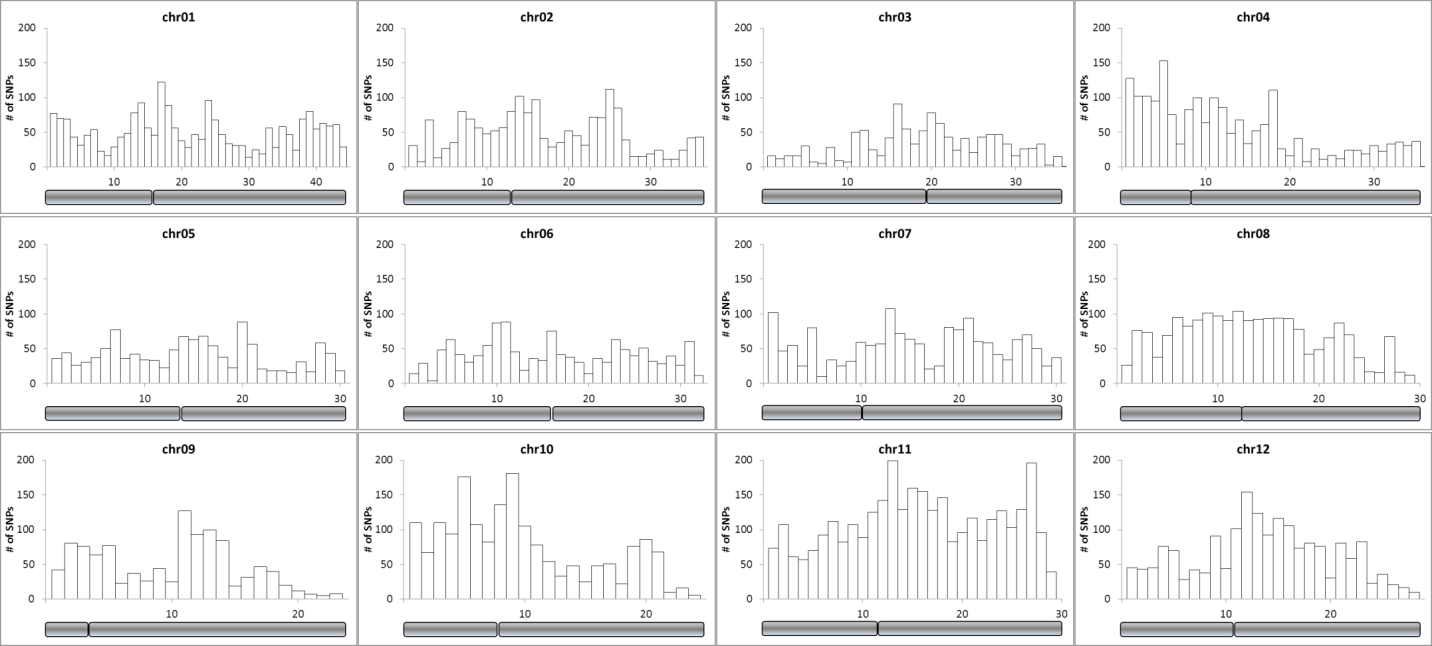


**Fig. S2 Chromosomal distribution of SNPs found in 45 California cultivars** The x-axis is the physical distance along a chromosome, split into 1-Mb windows. The triangle marker on the x-axis shows the position of the centromere of each chromosome. The y-axis is the number of SNPs in 1-Mb windows.
