# Supplementary material for: Identification of SNPs in Closely Related Temperate Japonica Rice Cultivars Using Restriction Enzyme-Phased Sequencing
Source: PLoS One. 2013 Mar 26;8(3):e60176. doi: 10.1371/journal.pone.0060176 (PMC3608622; doi:10.1371/journal.pone.0060176)
Supplement: Figure S3 — Population structure analysis using the STRUCTURE program. (DOCX) [file pone.0060176.s003.docx]

**Fig. S3 Population structure analysis using the STRUCTURE program** (A) Population structure plots (K = 2 to 10) (B) Probability of each k value being the true number of population (C) Adhoc statistic Δk which uses the second order of changes to identify K.
