# Supplementary material for: Identification of SNPs in Closely Related Temperate Japonica Rice Cultivars Using Restriction Enzyme-Phased Sequencing
Source: PLoS One. 2013 Mar 26;8(3):e60176. doi: 10.1371/journal.pone.0060176 (PMC3608622; doi:10.1371/journal.pone.0060176)
Supplement: Table S1 — List of California rice cultivars. (DOCX) [file pone.0060176.s004.docx]

Table S1 List of California rice cultivars

| **Cultivar** | **Group** | **Year** | **Pedigree** |
| --- | --- | --- | --- |
| Colusa | TMJ-S | 1917 | Chinese |
| Caloro | TMJ-S | 1921 | Early Wataribune |
| Calrose | TMJ-M | 1948 | Caloro/Calady*2 |
| CS-M3 | TMJ-M | 1971 | Smooth No.4-Calady40/Calrose |
| CS-S4 | TMJ-S | 1972 | Caloro/SmoothNo.3//Caloro/3/Caloro |
| M5 | TMJ-M | 1975 | CS-M3 natural mutation selections |
| S6 | TMJ-S | 1975 | Colusa/CS-M3 |
| Calrose76 | TMJ-M | 1976 | Induced mutant in Calrose |
| M7 | TMJ-M | 1978 | Calrose76/CS-M3 |
| M9 | TMJ-M | 1978 | IR-8/CS-M3*2//10-7*2 |
| Calmochi-201 | TMJ-S | 1979 | Mutant of S6 |
| L-201 | TRJ-L | 1979 | CI9701/3/R134-1/R48-257//R50-11 |
| M-101 | TMJ-M | 1979 | CS-M3/Calrose76//D31 |
| M-301 | TMJ-M | 1980 | Calrose76/CS-M3//M5 |
| S-201 | TMJ-S | 1980 | Calrose76/CS-M3//S6 |
| Calmochi-202 | TMJ-S | 1981 | R57-362-4/D51//Calmochi-201 |
| M-302 | TMJ-M | 1981 | Calrose76/CS-M3//M5 |
| M-401 | TMJ-M | 1981 | Induced mutant of Terso |
| M-201 | TMJ-M | 1982 | Terso/3/IR-8/CS-M3*2//Kokuhorose |
| L-202 | TRJ-L | 1984 | PI723761/PI7232278//L-201 |
| Calmochi-101 | TMJ-S | 1985 | Tatsumimochi//M7/S6 |
| M-202 | TMJ-M | 1985 | IR-8/CS-M3*2//10-7*2/3/M-101 |
| A-301 | TRJ-L | 1987 | IR-22/R48-257//5915C35-8/3/Della |
| M-102 | TMJ-M | 1987 | M-201/M-101 |
| M-203 | TMJ-M | 1988 | Mutant of M-401 |
| S-101 | TMJ-S | 1988 | 70-6526//R26/Toyohikari/3/M7/74-Y-89//SD7/73-221 |
| M-103 | TMJ-M | 1989 | 78-D-18347/M-302 |
| S-301 | TMJ-S | 1990 | SD7/73-221/M7P-1/3/M7P-5 |
| L-203 | TRJ-L | 1991 | L-202/83-Y-45 |
| M-204 | TMJ-M | 1994 | M-201/M7/3/M7//ESD7-3/Kokuhorose |
| A-201 | TRJ-L | 1996 | L-202/PI457920//L-202 |
| L-204 | TRJ-L | 1996 | Lemont//Tainung-sen-yu2414/L-201 |
| S-102 | TMJ-S | 1996 | Calpearl/Calmochi-101//Calpearl |
| Calhikari-201 | TMJ-S | 1999 | Koshihikari/(Koshihikari/S-101)*2 |
| Calmati-201 | TRJ-L | 1999 | 82-Y-51/83-Y-45//L-202/PI373938/3/83Y-45/PI457918 |
| L-205 | TRJ-L | 1999 | M7/R660//M7/R1588/3/82-Y-52/4/Rexmont/83-Y-45 |
| M-402 | TMJ-M | 1999 | Kokuhorose/4/M7*2/M9//M7/3/M-401/Kokuhorose |
| M-104 | TMJ-M | 2000 | M-103/6/F1(M-102/4/M-201/3/M7/M9//M7/5/M-103 |
| M-205 | TMJ-M | 2000 | M-201/M7//M-201/3/M-202 |
| M-206 | TMJ-M | 2003 | S-301/M-204 |
| M-207 | TMJ-M | 2005 | F1(Lafitte/5/Calpearl/3/M7/M9//M7/4/Calperarl)/6/M-202 |
| Calamylow-201 | TMJ-S | 2006 | Induced mutant of Calhikari-201 |
| Calmati-202 | TRJ-L | 2006 | A-201/9543483(Calmati-201sib) |
| L-206 | TRJ-L | 2006 | L-203/4/Lemont/3/R1588/L‑201//R1588/Labelle |
| M-208 | TMJ-M | 2006 | M-401/3/Mercury//Mercury/Koshihikari/4/M-204 |
