# Supplementary material for: Identification of SNPs in Closely Related Temperate Japonica Rice Cultivars Using Restriction Enzyme-Phased Sequencing
Source: PLoS One. 2013 Mar 26;8(3):e60176. doi: 10.1371/journal.pone.0060176 (PMC3608622; doi:10.1371/journal.pone.0060176)
Supplement: Table S2 — Multiplexing barcodes and number of raw sequence reads. (DOCX) [file pone.0060176.s005.docx]

Table S2 Multiplexing barcodes and number of raw sequence reads

|  | LabChip | | | AMPure | | | Total Reads | |
| --- | --- | --- | --- | --- | --- | --- | --- | --- |
| Cultivar | Barcode | Seq. Reads | % | Barcode | Seq. Reads | % | Seq. Reads | % |
| Colusa | AAACA | 7,168,750 | 4.5% | GACTT | 1,741,240 | 2.5% | 8,909,990 | 3.9% |
| Caloro | AAATC | 4,717,500 | 3.0% | GAGCA | 854,159 | 1.2% | 5,571,659 | 2.4% |
| Calrose | AACAT | 5,133,750 | 3.2% | GATAC | 1,284,716 | 1.8% | 6,418,466 | 2.8% |
| CS-M3 | AAGAG | 4,301,250 | 2.7% | GATGA | 2,012,187 | 2.9% | 6,313,437 | 2.8% |
| CS-S4 | AAGCC | 3,145,000 | 2.0% | GCAAA | 1,012,417 | 1.5% | 4,157,417 | 1.8% |
| M5 | AAGTT | 7,515,625 | 4.7% | GCCAT | 1,602,015 | 2.3% | 9,117,640 | 4.0% |
| S6 | AATGC | 5,503,750 | 3.4% | GCCTA | 955,740 | 1.4% | 6,459,490 | 2.8% |
| Calrose76 | ACACT | 3,908,125 | 2.4% | GCGTT | 1,864,130 | 2.7% | 5,772,255 | 2.5% |
| M7 | ACCCC | 2,497,500 | 1.6% | GCTCC | 592,681 | 0.8% | 3,090,181 | 1.3% |
| M9 | ACCGG | 3,306,875 | 2.1% | GCTGG | 1,082,994 | 1.6% | 4,389,869 | 1.9% |
| Calmochi-201 | ACGCA | 2,289,375 | 1.4% | GGAAC | 687,973 | 1.0% | 2,977,348 | 1.3% |
| L-201 | ACGTG | 7,215,000 | 4.5% | GGATG | 1,033,291 | 1.5% | 8,248,291 | 3.6% |
| M-101 | ACTAA | 5,989,375 | 3.7% | GGCAG | 1,295,413 | 1.9% | 7,284,788 | 3.2% |
| M-301 | ACTCG | 5,156,875 | 3.2% | GGGAA | 1,772,085 | 2.5% | 6,928,960 | 3.0% |
| S-201 | ACTGT | 7,053,125 | 4.4% | GGTGT | 1,676,927 | 2.4% | 8,730,052 | 3.8% |
| Calmochi-202 | AGCGA | 2,705,625 | 1.7% | GTACC | 1,416,659 | 2.0% | 4,122,284 | 1.8% |
| M-302 | AGCTT | 4,717,500 | 3.0% | GTAGT | 2,282,746 | 3.3% | 7,000,246 | 3.0% |
| M-401 | AGGAC | 1,364,375 | 0.9% | GTATA | 2,273,458 | 3.3% | 3,637,833 | 1.6% |
| M-201 | AGTCA | 601,250 | 0.4% | GTCAA | 1,490,116 | 2.1% | 2,091,366 | 0.9% |
| L-202 | AGTGG | 1,133,125 | 0.7% | GTCCT | 1,032,817 | 1.5% | 2,165,942 | 0.9% |
| Calmochi-101 | ATACG | 578,125 | 0.4% | GTCTG | 1,320,035 | 1.9% | 1,898,160 | 0.8% |
| M-202 | ATAGC | 855,625 | 0.5% | GTGAG | 1,503,594 | 2.2% | 2,359,219 | 1.0% |
| A-301 | ATCAC | 2,335,625 | 1.5% | GTGGA | 2,398,689 | 3.4% | 4,734,314 | 2.1% |
| M-102 | ATGTA | 4,324,375 | 2.7% | GTTAT | 2,636,872 | 3.8% | 6,961,247 | 3.0% |
| M-203 | ATTCC | 2,497,500 | 1.6% | GTTCA | 2,421,789 | 3.5% | 4,919,289 | 2.1% |
| S-101 | ATTGA | 2,844,375 | 1.8% | GTTTC | 1,612,831 | 2.3% | 4,457,206 | 1.9% |
| M-103 | CAAAA | 462,500 | 0.3% | TAAGA | 1,674,215 | 2.4% | 2,136,715 | 0.9% |
| S-301 | CAATT | 1,711,250 | 1.1% | TACTG | 372,250 | 0.5% | 2,083,500 | 0.9% |
| L-203 | CACCC | 1,618,750 | 1.0% | TAGCT | 590,195 | 0.8% | 2,208,945 | 1.0% |
| M-204 | CATCT | 2,728,750 | 1.7% | TATAG | 2,328,159 | 3.3% | 5,056,909 | 2.2% |
| A-201 | CATTC | 6,382,500 | 4.0% | TATCC | 2,155,574 | 3.1% | 8,538,074 | 3.7% |
| L-204 | CCCCA | 1,803,750 | 1.1% | TCATT | 2,150,884 | 3.1% | 3,954,634 | 1.7% |
| S-102 | CCCTT | 2,821,250 | 1.8% | TCCAG | 1,769,153 | 2.5% | 4,590,403 | 2.0% |
| Calhikari-201 | CCGAG | 2,566,875 | 1.6% | TCCCT | 1,234,352 | 1.8% | 3,801,227 | 1.7% |
| Calmati-201 | CGAAT | 2,936,875 | 1.8% | TCGAC | 430,818 | 0.6% | 3,367,693 | 1.5% |
| L-205 | CGAGG | 786,250 | 0.5% | TCGCG | 167,824 | 0.2% | 954,074 | 0.4% |
| M-402 | CGATC | 1,641,875 | 1.0% | TCGGA | 521,025 | 0.7% | 2,162,900 | 0.9% |
| M-104 | CGCAC | 1,711,250 | 1.1% | TGAAG | 1,264,895 | 1.8% | 2,976,145 | 1.3% |
| M-205 | CGCGT | 1,410,625 | 0.9% | TGACC | 932,411 | 1.3% | 2,343,036 | 1.0% |
| M-206 | CGGTT | 2,566,875 | 1.6% | TGATA | 2,909,909 | 4.2% | 5,476,784 | 2.4% |
| M-207 | CGTTG | 1,757,500 | 1.1% | TGGAT | 2,437,050 | 3.5% | 4,194,550 | 1.8% |
| Calamylow-201 | CTAAG | 1,965,625 | 1.2% | TGGTC | 1,913,433 | 2.7% | 3,879,058 | 1.7% |
| Calmati-202 | CTCGA | 670,625 | 0.4% | TGTCG | 1,221,182 | 1.7% | 1,891,807 | 0.8% |
| L-206 | CTCTC | 2,728,750 | 1.7% | TGTGC | 1,838,013 | 2.6% | 4,566,763 | 2.0% |
| M-208 | CTGCC | 1,410,625 | 0.9% | TGTTT | 4,019,397 | 5.8% | 5,430,022 | 2.4% |
| Sum |  | 138,541,875 |  |  | 69788313 |  | 20,833,0188 |  |
