# Supplementary material for: Identification of SNPs in Closely Related Temperate Japonica Rice Cultivars Using Restriction Enzyme-Phased Sequencing
Source: PLoS One. 2013 Mar 26;8(3):e60176. doi: 10.1371/journal.pone.0060176 (PMC3608622; doi:10.1371/journal.pone.0060176)
Supplement: Table S4 — Primer sequences for CAPS marker. (DOCX) [file pone.0060176.s007.docx]

Table S4 Primer sequences for CAPS marker

| Name | Sequence | Tm | Amplicon (bp) | Size of fragments after NlaII cut | |
| --- | --- | --- | --- | --- | --- |
|  |  |  |  | REF | ALT |
| CAPS_01L | CGAAACCTCTCCCACTGACTA | 59.3 | 348 | **(146,36)**, 225 | **(182)**, 225 |
| CAPS_01R | GGACCAAGGAGACCGAGTTTA | 60.5 |  |  |  |
| CAPS_02L | TGCATGATTCTGCCAGTGTTA | 60.3 | 374 | **(223, 37)**, 22, 74 | **(260)**, 22, 74 |
| CAPS_02R | CCAATATGTCGAGGCTCATGT | 60.0 |  |  |  |
| CAPS_03L | GAGACGCTGATGCTTGGTACT | 59.5 | 367 | **(107, 34)**, 16, 70, 85, 29 | **(141)**, 16, 70, 85, 29 |
| CAPS_03R | CTCCTCCATCTCATGCTTTTG | 59.8 |  |  |  |
| CAPS_07L | GTGTGAGGCATATGGTGTCG | 60.0 | 348 | **(162, 34)**, 22, 118 | **(196)**, 22, 118 |
| CAPS_07R | CACACCCTACCTCAAAGGACA | 60.0 |  |  |  |
| CAPS_12L | CTTGCTTCTTTTGGTGCTACG | 60.1 | 360 | **(210, 150)** | **(360)** |
| CAPS_12R | TCAGATCGTAGATCGGATGAG | 57.9 |  |  |  |
