# Supplementary material for: Identification of SNPs in Closely Related Temperate Japonica Rice Cultivars Using Restriction Enzyme-Phased Sequencing
Source: PLoS One. 2013 Mar 26;8(3):e60176. doi: 10.1371/journal.pone.0060176 (PMC3608622; doi:10.1371/journal.pone.0060176)
Supplement: Table S5 — Comparison of genotype results between RESCAN and CAPS marker. (DOCX) [file pone.0060176.s008.docx]

Table S5 Comparison of genotype results between RESCAN and CAPS marker.

| Cultivars | chr01 | | chr02 | | chr03 | | chr07 | | chr12 | |
| --- | --- | --- | --- | --- | --- | --- | --- | --- | --- | --- |
|  | SNP | CAPS | SNP | CAPS | SNP | CAPS | SNP | CAPS | SNP | CAPS |
| Colusa | 0 | 0 | 0 | 0 | 0 | 0 | 0 | 0 | 0 | 0 |
| Caloro | 0 | 0 | 0 | 0 | 0 | 0 | 0 | 0 | 0 | 0 |
| Calrose | 0 | 0 | 0 | 0 | 0 | 0 | 0 | 0 | - | - |
| CS-M3 | 0 | 0 | 0 | 0 | 0 | 0 | 1 | 1 | 1 | 1 |
| CS-S4 | 0 | 0 | 0 | 0 | 0 | 0 | 0 | 0 | 0 | 0 |
| M5 | 0 | 0 | 0 | 0 | 0 | 0 | 1 | 1 | 1 | 1 |
| S6 | 0 | 0 | 0 | 0 | 0 | 0 | 1 | 1 | 0 | 0 |
| Calrose 76 | 0 | 0 | 0 | 0 | 0 | 0 | 0 | 0 | 1 | 1 |
| M7 | - | 1 | 0 | 0 | - | 0 | 1 | 1 | 1 | 1 |
| M9 | 1 | 1 | 0 | 0 | 0 | 0 | 1 | 1 | 1 | 1 |
| Calmochi-201 | 0 | 0 | 0 | 0 | 0 | 0 | 1 | 1 | - | 1 |
| L-201 | 0 | 0 | 1 | 1 | 1 | 1 | 0 | 0 | 1 | 1 |
| M-101 | 0 | 0 | 0 | 0 | 0 | 0 | 0 | 0 | 0 | 0 |
| M-301 | 0 | 0 | 0 | 0 | 0 | 0 | 0 | 0 | 1 | 1 |
| S-201 | 0 | 0 | 0 | 0 | 0 | 0 | 0 | 0 | 1 | 1 |
| Calmochi-202 | 0 | 0 | 0 | 0 | 0 | 0 | 1 | 1 | 0 | 0 |
| M-302 | 0 | 0 | 0 | 0 | 0 | 0 | 1 | 1 | 1 | 1 |
| M-401 | - | 1 | 0 | 0 | 0 | 0 | 0 | 0 | - | 0 |
| M-201 | 1 | 1 | 0 | 0 | - | 0 | 1 | 0 | - | 0 |
| L-202 | - | 1 | 0 | 0 | - | 1 | 0 | 0 | 1 | 1 |
| Calmochi-101 | - | 1 | 0 | 0 | - | 0 | 0 | 0 | - | 0 |
| M-202 | - | 0 | 0 | 0 | 0 | 0 | 1 | 1 | 1 | 1 |
| A-301 | 1 | 1 | - | 1 | 1 | 1 | 0 | 0 | 1 | 0 |
| M-102 | 0 | 0 | 0 | 0 | 0 | 0 | 1 | 0 | 0 | 0 |
| M-203 | - | 0 | 0 | 0 | 0 | 0 | 0 | 0 | 1 | 0 |
| S-101 | 0 | 0 | 0 | 0 | 0 | 0 | 1 | 1 | 0 | 0 |
| M-103 | - | 0 | - | 0 | - | 0 | 1 | 1 | - | 0 |
| S-301 | - | 0 | 0 | 0 | 0 | 0 | 0 | 0 | - | 1 |
| L-203 | - | 1 | 0 | 0 | - | 1 | 0 | 0 | 1 | 1 |
| M-204 | 1 | 1 | 0 | 0 | 0 | 0 | 1 | 1 | 1 | 1 |
| A-201 | 1 | 1 | 0 | 0 | 1 | 1 | 0 | 0 | 1 | 1 |
| L-204 | - | 1 | 0 | 0 | - | 1 | 0 | 0 | 1 | 1 |
| S-102 | 0 | 0 | 0 | 0 | 0 | 0 | 0 | 0 | 0 | 0 |
| Calhikari-201 | 0 | 0 | 0 | 0 | 0 | 0 | 0 | 0 | 0 | 0 |
| Calmati-201 | 1 | 1 | 1 | 1 | 0 | 0 | 0 | 0 | 1 | 1 |
| L-205 | - | 1 | 0 | 0 | - | 1 | 0 | 0 | - | 1 |
| M-402 | - | 0 | 0 | 0 | 0 | 0 | 1 | 1 | 1 | 1 |
| M-104 | - | 0 | 0 | 0 | 0 | 0 | 1 | 1 | 1 | 1 |
| M-205 | - | 0 | 0 | 0 | 0 | 0 | 1 | 1 | - | 0 |
| M-206 | 0 | 0 | 0 | 0 | 0 | 0 | 1 | 1 | 1 | 1 |
| M-207 | - | 0 | 0 | 0 | 0 | 0 | 1 | 1 | 0 | 0 |
| Calamylow-201 | - | 1 | 0 | 0 | 0 | 0 | 0 | 0 | 0 | 0 |
| Calmati-202 | - | 1 | - | 0 | 0 | 0 | 1 | 1 | - | 0 |
| L-206 | 1 | 1 | 0 | 1 | - | 1 | 0 | 0 | 1 | 1 |
| M-208 | - | 0 | 0 | 0 | 0 | 0 | 1 | 1 | 1 | 1 |
| Nipponbare | 0 | 0 | 0 | 0 | 0 | 0 | 0 | 0 | 0 | 0 |

* Genotypes were represented based on Nipponbare as a reference: Nipponbare (0) and different allele (1). Discrepancy between RESCAN and CAPS marker were highlighted.
